# Astrocyte-specific regulation of hMeCP2 expression in *Drosophila*

**DOI:** 10.1242/bio.20149092

**Published:** 2014-10-10

**Authors:** David L. Hess-Homeier, Chia-Yu Fan, Tarun Gupta, Ann-Shyn Chiang, Sarah J. Certel

**Affiliations:** 1 Division of Biological Sciences, The University of Montana, Missoula, MT 59812, USA; 2 Neuroscience Graduate Program, The University of Montana, Missoula, MT 59812, USA; 3 Biomedical Technology and Device Research Laboratories, Industrial Technology Research Institute, Hsinchu 31040, Taiwan; 4 Brain Research Center, National Tsing Hua University, Hsinchu 30013, Taiwan

**Keywords:** MeCP2, Rett Syndrome, Sleep, Astrocytes, *Drosophila*

## Abstract

Alterations in the expression of Methyl-CpG-binding protein 2 (MeCP2) either by mutations or gene duplication leads to a wide spectrum of neurodevelopmental disorders including Rett Syndrome and MeCP2 duplication disorder. Common features of Rett Syndrome (RTT), MeCP2 duplication disorder, and neuropsychiatric disorders indicate that even moderate changes in MeCP2 protein levels result in functional and structural cell abnormalities. In this study, we investigated two areas of MeCP2 pathophysiology using *Drosophila* as a model system: the effects of MeCP2 glial gain-of-function activity on circuits controlling sleep behavior, and the cell-type specific regulation of MeCP2 expression. In this study, we first examined the effects of elevated MeCP2 levels on microcircuits by expressing human MeCP2 (hMeCP2) in astrocytes and distinct subsets of amine neurons including dopamine and octopamine (OA) neurons. Depending on the cell-type, hMeCP2 expression reduced sleep levels, altered daytime/nighttime sleep patterns, and generated sleep maintenance deficits. Second, we identified a 498 base pair region of the MeCP2e2 isoform that is targeted for regulation in distinct subsets of astrocytes. Levels of the full-length hMeCP2e2 and mutant RTT R106W protein decreased in astrocytes in a temporally and spatially regulated manner. In contrast, expression of the deletion Δ166 hMeCP2 protein was not altered in the entire astrocyte population. qPCR experiments revealed a reduction in full-length *hMeCP2e2* transcript levels suggesting transgenic hMeCP2 expression is regulated at the transcriptional level. Given the phenotypic complexities that are caused by alterations in MeCP2 levels, our results provide insight into distinct cellular mechanisms that control MeCP2 expression and link microcircuit abnormalities with defined behavioral deficits.

## INTRODUCTION

Methyl-CpG-binding protein 2 (MeCP2) is one of the most dosage-sensitive genes involved in neuronal functional integrity. It is thought to perform a complex role in the cell including coordinating chromatin remodeling, RNA processing, and promoter repression or activation, depending on the molecular context (Adkins and Georgel, 2011; Na and Monteggia, 2011; Samaco and Neul, 2011). Altered levels of MeCP2 either through loss-of-function mutations (Rett Syndrome (RTT)) or increased protein levels due to gene duplication or triplication (MeCP2 Duplication Syndrome) (Amir et al., 1999; Ramocki et al., 2010), results in dramatic phenotypes including mild to profound intellectual disabilities, motor dysfunction, features of autism, stereotyped hand movements, recurrent respiratory infections, and sleep disturbances, and are known collectively as MeCP2 spectrum disorders (MSD). Overexpression of MeCP2 via transgenic mouse studies or in patients with MeCP2 Duplication Syndrome link core behavioral aspects of Autism Spectrum Disorder (ASD) directly to MeCP2 cellular effects and expand the clinical relevance of understanding MeCP2 function (Coutinho et al., 2007; Neul, 2012; Ramocki et al., 2009). In addition, alterations in *MeCP2* expression levels may manifest in neuropsychiatric conditions including juvenile onset schizophrenia and bipolar disease with mental retardation depending on the type of mutation or the pattern of X chromosome inactivation (Gonzales and LaSalle, 2010; Ramocki et al., 2009). These findings indicate that maintaining MeCP2 levels in a narrow range is a critical component of proper nervous system development and overall brain function.

Levels of MeCP2 are regulated spatio-temporally in the human brain with each cell-type expressing a distinct amount (Han et al., 2013; Shibayama et al., 2004; Skene et al., 2010). MeCP2 expression is highest in neurons yet recent studies indicate wildtype levels in glia are also essential for maintaining cellular and network homeostasis. Cultured MeCP2-deficient astrocytes exhibit striking differences in gene expression, growth rate, cytotoxic effects, microtubule dynamics, and glutamate (Glu) clearance that may influence the onset and progression of RTT (Okabe et al., 2012). The significance of these cellular and structural consequences of altered MeCP2 glial expression is apparent as transplantation of wildtype microglia can arrest multiple aspects of disease pathology exhibited in a MeCP2-null mouse (Derecki et al., 2012). Although just as clinically relevant, the consequences of high MeCP2 levels in glia are less understood.

In this study, we used *Drosophila* as a model system to first examine the effects of elevated MeCP2 levels on sleep-related microcircuits by expressing human MeCP2e2 (hMeCP2) in astrocytes and distinct subsets of amine neurons including dopamine and octopamine (OA) neurons. The basic cellular properties of human MeCP2 are maintained upon expression in *Drosophila* including chromatin association, interactions with human chromatin remodeling gene homologs, and post-translational modifications (Cukier et al., 2008; Vonhoff et al., 2012) which suggests, on a molecular basis, at least a subset of hMeCP2 functional capabilities are preserved. In addition, proteins containing a methyl-CpG-binding domain (MBD) are conserved from flies to humans (Hendrich and Tweedie, 2003; Hu et al., 2006) and hMeCP2-expression causes defects in neuronal structure and motor behavior (Vonhoff et al., 2012). We examined the sleep behavior of males expressing hMeCP2 in astrocytes and for comparison, in distinct subsets of amine neurons including dopamine and octopamine (OA) neurons. hMeCP2 expression reduced sleep levels, altered daytime/nighttime sleep patterns, and generated difficulties in maintaining sleep and initiating sleep. Each parameter was uniquely affected depending on the cell-type expressing hMeCP2.

As the number of neuronal and non-neuronal cell-types and molecular targets of MeCP2 increases, the consideration of potentially regulating MeCP2 expression itself becomes of great interest. In a second set of experiments examining the transgenic expression of MeCP2, we found full-length hMeCP2 (hMeCP2^FL^) and RTT R106W protein levels decreased in glial subsets in a temporally and spatially regulated manner. This result was specific to astrocytes as hMeCP2 levels were not altered in amine neurons. Further analyses revealed that astrocytes expressing the *hMeCP2^Δ166^* allele maintain detectable protein levels indicating this 498 base pair region contains a site that may be targeted by a cell-specific endogenous factor. Quantification of glial number indicated hMeCP2-expressing astrocytes are not dying but rather the reduction of hMeCP2 levels is via a transcriptional mechanism. Taken together, our results address aspects of MeCP2 cell-specific function and regulation by demonstrating astrocyte expression alters the output of neuronal sleep-related circuitry and hMeCP2 protein levels are regulated through a novel site located in the n-terminus.

## RESULTS

### Astrocyte-expression of hMeCP2 alters sleep parameters

**Fig. 1.**
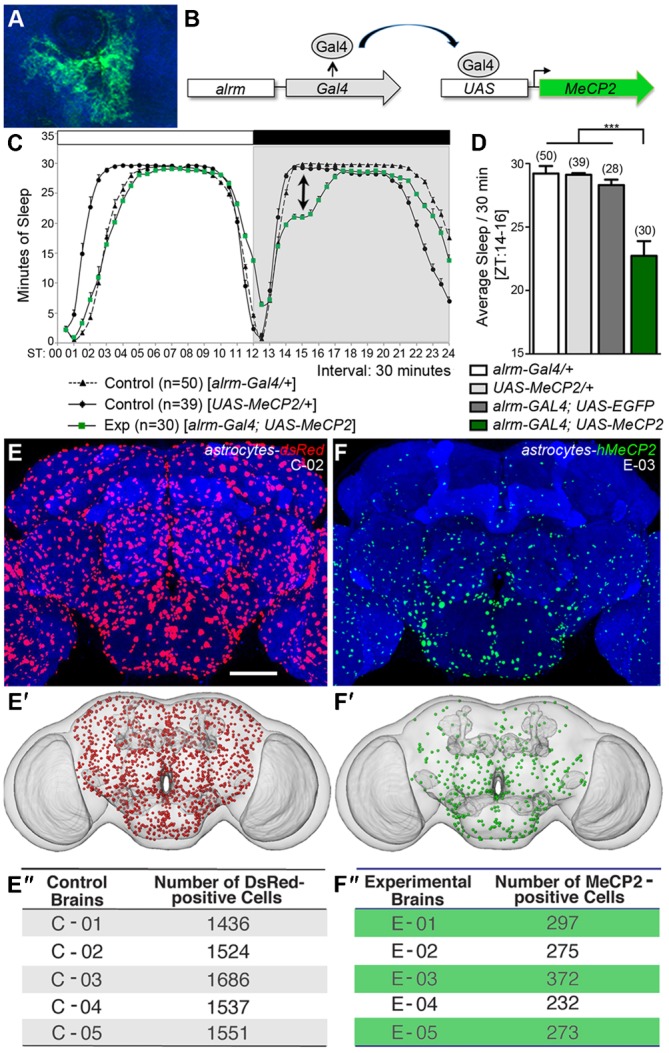
Sleep and expression levels are altered in adult flies expressing hMeCP2 in astrocytes. (A) An individual *Drosophila* astrocyte visualized by CD8:GFP expression in a *UAS-CD8:GFP/alrm-Gal4* adult brain. (B) The Gal4/UAS bipartite system drives transgenic hMeCP2 expression in astrocytes via *alrm-Gal4.* All *alrm-Gal4* progeny contained both the *alrm-Gal4#3* and *alrm-Gal4#2* transgenes. (C,D) The minutes of sleep over a 24 hour period covering a 10 day span from individual control males *(alrm-Gal4/+,alrm-Gal4/+, UAS-hMeCP2^FL^/+*, or *alrm-Gal4/+;alrm-Gal4/UAS-EGFP*) and experimental (*alrm-Gal4/+; alrm-Gal4/UAS-hMeCP2^FL^)* males. (C) Eduction graph displaying 12 hour night (black bar) and 12 hour day (white bar) periods in control and experimental males. (D) Males expressing hMeCP2^FL^ in astrocytes exhibited a decrease in sleep during the day-to-night transition and through hours 22–24 as compared to controls (F: 20.122, *P*<0.001, d = 1.458; one-way ANOVA of bootstrapped residuals; Nrep = 50,000; d  =  effect size; pairwise comparisons indicate significant differences in all control-experimental group pairs) (arrow in panel C). (E–E″) Astrocytes in control adult brain expressing nuclear dsRed in an *alrm-Gal4/UAS-dsRed;alrm-Gal4/+* progeny. (E′) Nuclear-labeled astrocytes from the central brain projected onto a standard *Drosophila* brain representation. (E″) Quantification of astrocytes located in the central brain region in control brains (*alrm-Gal4/UAS-dsRed;alrm-Gal4/+*). (F–F″) Astrocytes expressing hMeCP2^FL^ in experimental *alrm-Gal4/+;alrm-Gal4/UAS-hMeCP2^FL^* progeny. hMeCP2 is detected by immunofluorescence using a rabbit hMeCP2 antibody (green, Cell Signaling). (F′) Central brain hMeCP2^FL^-labeled astrocytes projected onto a standard *Drosophila* brain representation. (F″) Quantification of astrocytes expressing detectable hMeCP2^FL^ in *alrm-Gal4/+;alrm-Gal4/UAS-hMeCP2^FL^* experimental progeny. Scale bar represents 50 µm.

Reciprocal interactions between glia and neurons are essential for many critical brain functions and astrocytes, in particular, are involved in the control of multiple neuronal activities (Bekar et al., 2008; Fillenz et al., 1999; Perea and Araque, 2007). *Drosophila* astrocyte-like cells (termed cortex glia) exhibit similarities to their mammalian counterparts including morphologically as large, star-shaped cells with extensive processes that surround neuronal cell bodies (Fig. 1A), and functionally with the expression of conserved molecular markers such as the high-affinity excitatory amino acid transporters (EAATs) (Doherty et al., 2009; Freeman and Doherty, 2006; Soustelle et al., 2002). Recent studies indicate that the loss of MeCP2 in glia can exert a non-cell-autonomous negative effect on neighboring neurons (Lioy et al., 2011; Maezawa and Jin, 2010; Maezawa et al., 2009), however less is known about potential neuron functional changes due to elevated levels of glial MeCP2 expression. To test if astrocytic MeCP2 expression non-cell autonomously alters neuron function, we utilized *Drosophila* as an *in vivo* model system and examined sleep as a relevant behavioral representation of circuit dysfunction.

Sleep disorders are prevalent among children with MeCP2 spectrum disorders and are characterized by delays in the onset of sleep, alterations in total sleep, and frequent wakings resulting in fragmented sleep (Cortesi et al., 2010; Piazza et al., 1990; Souders et al., 2009; Young et al., 2007). In the broadest sense, sleep is defined as a period of inactivity accompanied by an increase in arousal threshold and, if disrupted, is followed by a period of sleep rebound (Hendricks and Sehgal, 2004; Huber et al., 2004). This definition is also applied to genetically simple organisms like *C. elegans* and *Drosophila*. Two critical aspects of sleep, the timing of sleep and the length/quality of sleep (Borbély, 1982) are also components of the fruit fly sleep-like state.

Using the Gal4-UAS targeted gene expression system and previously characterized transgenic lines (Cukier et al., 2008) (Fig. 1B), we asked if sleep circuitry output is altered in adults expressing hMeCP2 in astrocytes through the *astrocytic leucine-rich repeat molecule (alrm)-Gal4* line (Doherty et al., 2009) (*alrm-Gal4;alrm-Gal4/UAS-hMeCP2^FL^* as compared to individual control groups (*alrm-Gal4;alrm-Gal4/+* and *UAS-hMeCP2^FL^/+*) (Fig. 1C,D). Using a standard automated behavioral-based system (Ho and Sehgal, 2005), we quantified total sleep, sleep patterns, and sleep fragmentation. The total time spent in sleep during a 24-hour period was significantly reduced in flies expressing hMeCP2 through the *alrm-Gal4* line compared to controls with the reduction occurring immediately after the day:evening transition (Fig. 1C,D, ZT12-15; supplementary material Fig. S1A,B). However, hMeCP2 expression in astrocytes did not result in fragmented sleep bouts (supplementary material Fig. S1C), nor do experimental flies lose rhythmicity in constant dark (DD) conditions or alter indicators of clock function such as rhythmicity and period (Tau) (supplementary material Fig. S1D–F; Table S1). Males expressing hMeCP2 in octopamine and dopamine neurons also displayed temporally restricted sleep reductions, which occurred in separate distinct time frames (T.G. and S.J.C., unpublished observations). These results suggest the expression of hMeCP2 in astrocytes and neurons can induce distinct alterations in sleep circuitry function in a cell-type specific manner.

### hMeCP2 expression in reduced in a subset of *Drosophila* astrocytes

To verify the functionality of the individual genetic components, we examined the transgenic expression levels of hMeCP2 in amine neurons and astrocytes. hMeCP2 expression in adult octopamine or dopamine neurons did not change (supplementary material Fig. S2), however a significant reduction or absence in hMeCP2 protein levels in astrocytes was observed. Similar to the widespread distribution in the vertebrate brain, *Drosophila* astrocytes can be found throughout the adult CNS as visualized by expression of a nuclear-tagged red fluorescent protein (*UAS-dsRed*) driven through the *alrm-Gal4* driver (Fig. 1B,E). Results from individual preparations of were superimposed on a standard *Drosophila* brain (Fig. 1E′) and the number of dsRed-positive astrocytes averaged 1547 cells/brain ± 40 (± s.e.m.) (Fig. 1E″). When we utilized the same *astrocyte(alrm)-Gal4* driver to produce hMeCP2 in all astrocytes, hMeCP2 protein was detected by antibody labeling in only a small number of astrocytes (290±23 (± s.e.m.)) (Fig. 1F,F′,F″).

**Fig. 2.**
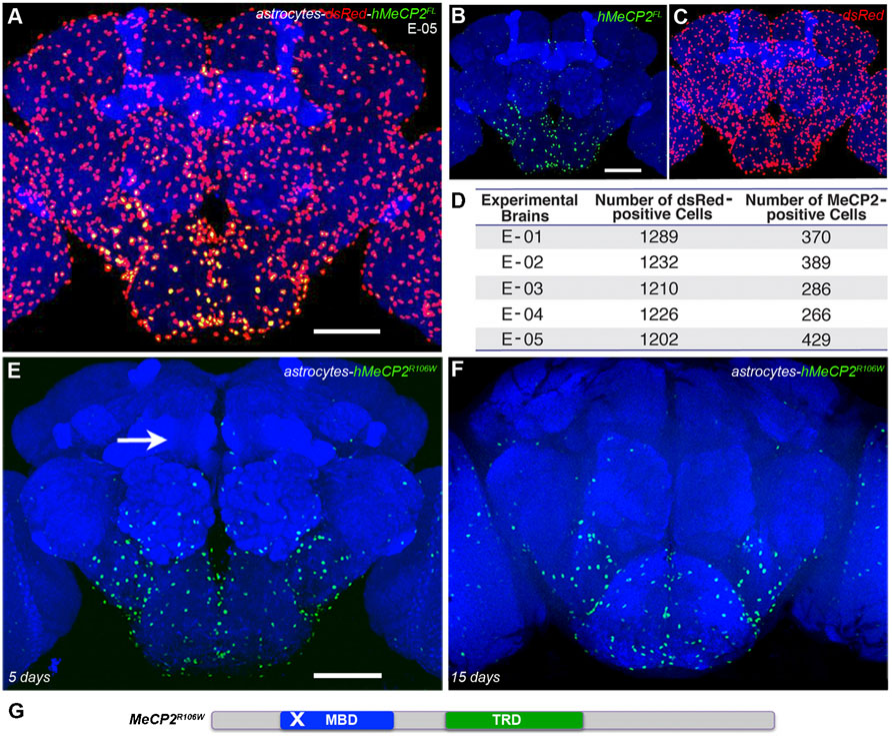
The *alrm-Gal4* population of astrocytes is present in hMeCP2^FL^-expressing brains. (A–C) Adult brain co-expressing hMeCP2^FL^ and dsRed in astrocytes (*UAS-dsRed/alrm-Gal4;alrm-Gal4/UAS-hMeCP2^FL^*). hMeCP2^FL^ expression is reduced or not detectable in a substantial number of astrocytes (B,D) that co-express dsRed (C,D). (D) Quantification of astrocytes expressing detectable hMeCP2^FL^ in *UAS-dsRed/alrm-Gal4;alrm-Gal4/UAS-hMeCP2^FL^* progeny. Scale bars represent 50 µm. (E–G) hMeCP2^R106W^ expression is reduced or not detectable in *alrm-Gal4* expressing astrocytes in a large part of the adult brain including the protocerebrum (arrow) at 5 (E) or 15 (F) days post-eclosion. (G) Schematic representation of the single point mutation in the MBD of the RTT allele *hMeCP2^R106W^*.

Transgenically all astrocytes should be capable of expressing hMeCP2 in the same manner as the dsRed reporter. To determine if astrocytes that have the competence to express hMeCP2 are still present, progeny carrying the *dsRed* reporter, the *hMeCP2* transgene, and the *alrm-Gal4* driver were generated. The full complement of dsRed-positive astrocytes is visible in *UAS-dsRed;alrm-Gal4;alrm-Gal4/UAS-hMeCP2^FL^* adult brains (Fig. 2A,C), however hMeCP2 protein is not detectable in the majority of astrocytes in regions including the protocerebrum, mushroom bodies, and antennal and optic lobes (Fig. 2A,B). Quantification of cell number indicates 1232±15 (± s.e.m.) astrocytes expressing dsRed are present and approximately one-fourth co-express detectable levels of hMeCP2 (348±31 (± s.e.m.)) (Fig. 2D). The reduction in hMeCP2-positive cells does not occur in a uniform manner; rather detectable hMeCP2 levels are consistently visible in astrocytes within the suboesophageal ganglion (SOG) a region that receives taste and contact pheromonal input (Miyazaki and Ito, 2010; Stocker, 1994).

To verify that the absence of hMeCP2-expression is not due to insufficient antibody penetration, we substituted the *UAS-dsRed* transgene with a *UAS-nuclearLacZ* transgene *(alrm-Gal4;UAS-nucLacZ/UAS-hMeCP2^FL^)*. Using antibodies against β-galactosidase (β-gal) and MeCP2, lacZ production was easily visible throughout the *Drosophila* central brain even in astrocytes without detectable hMeCP2 expression (supplementary material Fig. S3). The absence of detectable hMeCP2 protein is not due to a limiting amount of the Gal4 transcriptional activator as the lack or reduction of hMeCP2 is visible in progeny with one or two copies of the *alrm-Gal4* driver (supplementary material Fig. S3). At this point, our data indicates that despite the functionality of the *Gal4* driver, hMeCP2 protein is absent or reduced in subsets of astrocytes that contain the transgenic constructs to express hMeCP2. Our control experiments provide reproducible quantification of glial number and identification of the entire astrocyte population with either a fluorescent or antibody-visualized protein. With these results, our focus shifted from examining the potential cellular or circuitry changes as a consequence of hMeCP2 expression to investigating cell-specific regulation of hMeCP2.

### RTT hMeCP2^R106W^ expression is reduced in the same astrocyte subset

To rule out possible *UAS-hMeCP2^FL^* transgenic construct defects that may explain the loss of detectable hMeCP2 protein, we tested the RTT point mutation *hMeCP2^R106W^* transgenic line (*UAS-hMeCP2^R106W^*) (Cukier et al., 2008). The RTT R106W allele is a missense mutation within the methyl-CpG binding domain that greatly reduces the protein's ability to bind DNA (Fig. 2G) (Yusufzai and Wolffe, 2000). As predicted for a single amino acid change, expression of *hMeCP2^R106W^* in astrocytes (*alrm-Gal4/UAS-hMeCP2^R106W^,alrm-Gal4*) results in the same phenotype as full-length hMeCP2 expression, namely a reduction in detectable protein as assayed by antibody labeling in central brain astrocytes at 5 days post-eclosion (Fig. 2E). hMeCP2^R106W^ expression was maintained only in suboesophageal ganglia astrocytes in the aging brain (15 days post-eclosion, Fig. 2F).

### The reduction of hMeCP2 is developmentally regulated

In human brains, MeCP2 levels are relatively low during fetal stages and elevated in postnatal development (Balmer et al., 2003). To determine if the levels of transgenically produced hMeCP2 may be developmentally regulated in astrocytes in our *Drosophila* model, we first dissected the central nervous system from hMeCP2-expressing third instar larvae. The third instar larval period is the final stage of growth and feeding before the complex tissue reorganization of metamorphosis occurs during pupation (Hertweck, 1931; Power, 1947; Truman, 1990). Astrocytes within the central brain of third instar larvae exhibit co-localization of β-gal and hMeCP2 *(alrm-Gal4/UAS-nucLacZ;alrm-Gal4/UAS-hMeCP2^FL^)* (supplementary material Fig. S4A–C). An absence of hMeCP2 expression is observed in astrocytes located in the optic lobe (arrows, supplementary material Fig. S4A–C) suggesting differences exist in astrocyte lineages at this stage of larval development.

**Fig. 3.**
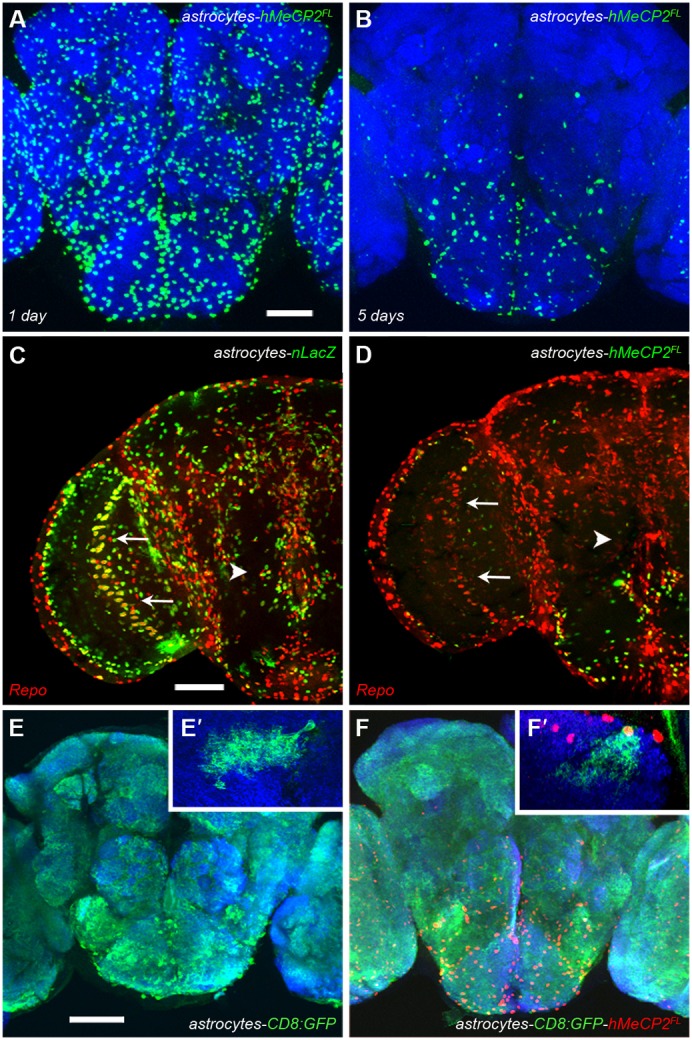
hMeCP2 expression is developmentally regulated and does not alter glial differentiation. (A) At 1 day post-eclosion, hMeCP2 expression is detected by immunofluorescence in the majority of *alrm-Gal4* expressing astrocytes. (B) At 5 days post-eclosion, hMeCP2 protein is reduced or not detectable in astrocytes located outside of the SOG region. (C) The transcription factor Repo is co-expressed with β-gal in optic lobe astrocytes (arrow) in *alrm-Gal4/UAS-nucLacZ;alrm-Gal4* progeny. (D) hMeCP2-expressing astrocytes maintain glial differentiation as assayed by Repo expression in the optic lobe (arrow) and the SOG (arrowhead) in the *alrm-Gal4/+;alrm-Gal4/UAS-hMeCP2^FL^* adult brain. Scale bars represent 50 µm. (E,E′) Confocal image of the large number of astrocytes labeled by a cell-membrane GFP reporter in the brain of a control *alrm-Gal4/+;alrm-Gal4/UAS-CD8:GFP* adult. (E′) Optical sections of a *hs-flp;alrm-Gal4/UAS->stop>CD8:GFP;alrm-Gal4/+* control brain at a high magnification showing a single GFP-expressing astrocyte. (F,F′) hMeCP2^FL^-expressing astrocytes labeled with the same cell-membrane GFP reporter in an adult *alrm-Gal4/UAS-CD8:GFP;alrm-Gal4/UAS-hMeCP2^FL^* brain five days post-eclosion. GFP-expressing astrocytes with or without detectable hMeCP2 (red nuclear expression) cover the entire brain as in control brains. (F′) Optical sections from a *hs-flp;alrm-Gal4/UAS->stop>CD8:GFP;alrm-Gal4/UAS-hMeCP2^FL^*brain at high magnification showing a single GFP and hMeCP2^FL^ -expressing astrocyte. The dense, fine processes observed in control astrocytes are present in astrocytes expressing hMeCP2^FL^. Scale bar represents 50 µm.

A developmental transition in hMeCP2 levels is also observed after larval stages. hMeCP2 expression is visible in all *alrm-Gal4* astrocytes throughout the brain of newly-eclosed adults (Fig. 3A). The striking reduction in detectable hMeCP2 expression as described in Figs 1 and 2 is visualized by 5 days post-eclosion with expression of hMeCP2 maintained in SOG astrocytes of aged (25 day post-eclosion, Fig. 3B) adults. Results from these experiments suggest the molecular composition of astrocytes regionally, i.e. within the optic lobe and temporally, i.e. at mature stages (adult and larval) include a gene product that regulates hMeCP2 at the transcriptional or translational level.

Due to the multiple proposed cellular targets of hMeCP2, we next examined if *alrm-Gal4;UAS-hMeCP2* cells continued to express markers consistent with glial differentiation. To identify subsets of glia, we used an antibody against the homeodomain protein, Repo (Reversed polarity). Repo plays a key role in glial development and diversification (Yuasa et al., 2003). Adult CNS glia (Awasaki et al., 2008) express Repo including astrocytes labeled in the *alrm-Gal4/UAS-nucLacZ;alrm-Gal4* adult brain (Fig. 3C,D). When the *UAS-nucLacZ* reporter is replaced with the *hMeCP2* transgene, we observe Repo-labeled astrocytes in the optic lobe (arrows, Fig. 3C) that are present but do not express detectable hMeCP2 protein (arrows, Fig. 3D). This result indicates that astrocytes previously producing hMeCP2 still maintain a glial state. In addition, we analyzed the morphology of individual astrocytes expressing GFP or astrocytes expressing GFP with hMeCP2 as distinct defects in dendritic structure are observed in *Drosophila* motor neurons expressing MeCP2 (Vonhoff et al., 2012). At this point, we are unable to compare the same astrocyte with or without hMeCP2 expression however, the morphology of individual astrocytic processes from the same brain region does not appear to be significantly altered by hMeCP2 expression (Fig. 3E–F″).

### hMeCP2^Δ166^ expression is detected in the entire *alrm-*expressing glia population

**Fig. 4.**
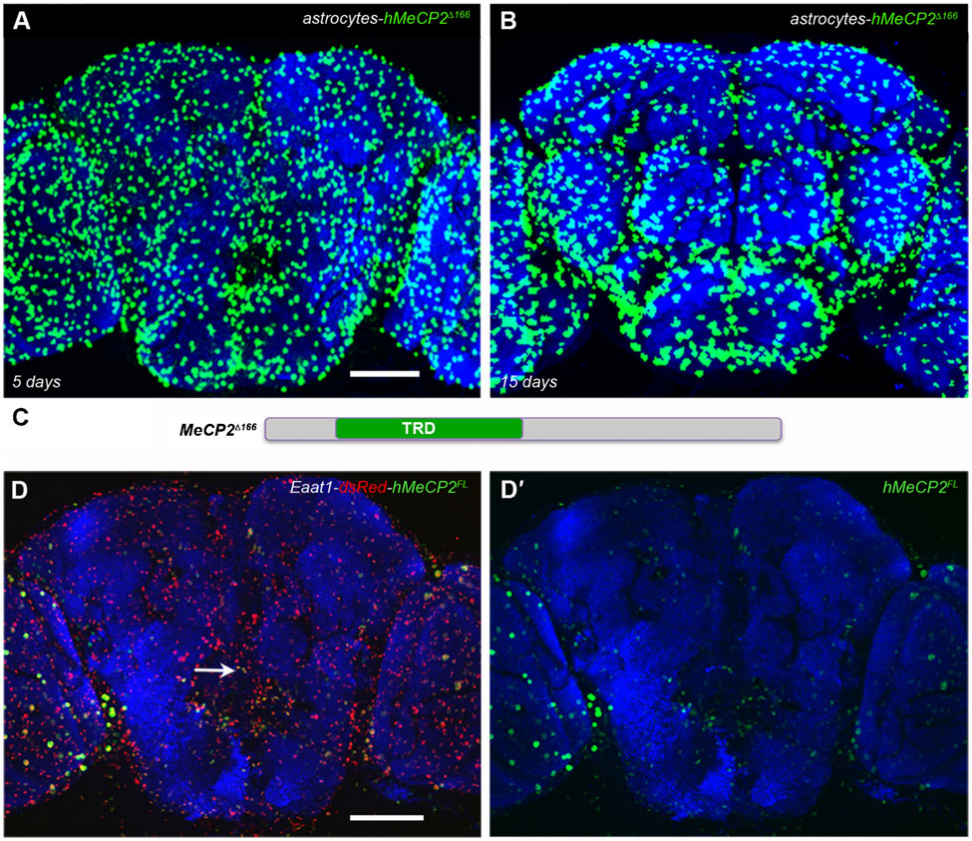
The hMeCP2^Δ166^ allele is expressed in all astrocytes. (A,B) hMeCP2^Δ166^ expression is detected in *alrm-Gal4* expressing astrocytes throughout the brain at 5 (A) or 15 (B) days post-eclosion (*alrm-Gal4/UAS- *hMeCP2*^Δ166^;alrm-Gal4/+*). (C) Schematic representation of the hMeCP2^Δ166^ allele containing a deletion of the N-terminus including the MBD. hMeCP2^Δ166^ expression is detected by immunofluorescence with the rabbit hMeCP2 antibody (green, Cell Signaling). (D,D′) Confocal images of two *UAS-dsRed;EAAT1-Gal4/UAS-hMeCP2^FL^* adult brains demonstrating hMeCP2FL protein is largely absent or reduced in the Eaat1 glial population. Co-expression of dsRed and hMeCP2^FL^ is maintained in a number of glia in the optic lobe (arrow) or around the antennal lobe. Scale bars represent 50 µm.

Using a third independent transgenic line, we expressed hMeCP2^Δ166^, which lacks the N-terminus including the MBD (Fig. 4C) (Cukier et al., 2008), via the *alrm-Gal4* driver. In contrast to the region-specific reduction in hMeCP2^FL^ or hMeCP2^R106W^ expression, the hMeCP2^Δ166^ protein is detected in the entire *alrm-Gal4* astrocyte population and is maintained in the aging brain (Fig. 4A,B). This result indicates that transgenic expression of hMeCP2 in all *alrm-Gal4* astrocytes is possible and suggests that the N-terminus contains a site that may be targeted by a cell-specific endogenous factor to transcriptionally or translationally regulate full-length hMeCP2 expression. Although astrocytes are often classified as a population of cells, glial subsets can display diverse molecular and functional profiles (Matthias et al., 2003; Nimmerjahn, 2009; Regan et al., 2007).

In particular, specific astrocyte populations take up and recycle the principal neurotransmitter in the brain, glutamate, by expressing high-affinity, sodium-dependent excitatory amino acid transporters (EAATs) (Lee and Pow, 2010; Zhou and Danbolt, 2013). EAAT1 and EAAT2 are expressed in discrete and partially overlapping subsets of differentiated glia in *Drosophila* (Rival et al., 2004; Soustelle et al., 2002). To demonstrate that a decrease in detectable hMeCP2 protein is observed in the EAAT1 subset of glial cells and that separate Gal4 lines are capable of producing the same hMeCP2 reduction phenotype, we drove hMeCP2^FL^ expression with the *EAAT1-Gal4* driver (Rival et al., 2004). When flies were generated to contain the *dsRed* reporter and the *hMeCP2* transgene together with the *EAAT1-Gal4* transgene (*UAS-dsRed;EAAT1-Gal4/UAS-hMeCP2^FL^)*, we observed a large complement of dsRed-positive astrocytes, however hMeCP2 protein is detectable at very low levels or absent in the majority of EAAT1-identified astrocytes (Fig. 4D,D′). In addition, a reduction or absence of hMeCP2 protein is also observed in a subset of inner-optic chiasm giant glia cells located in the larval optic lobe (supplementary material Fig. S5). Taken together, these experiments demonstrate that combinations of multiple Gal4 drivers and *UAS-MeCP2* lines result in the same reduction of hMeCP2 protein expression phenotype.

### Transcript levels of transgenic hMeCP2^FL^ are reduced

**Fig. 5.**
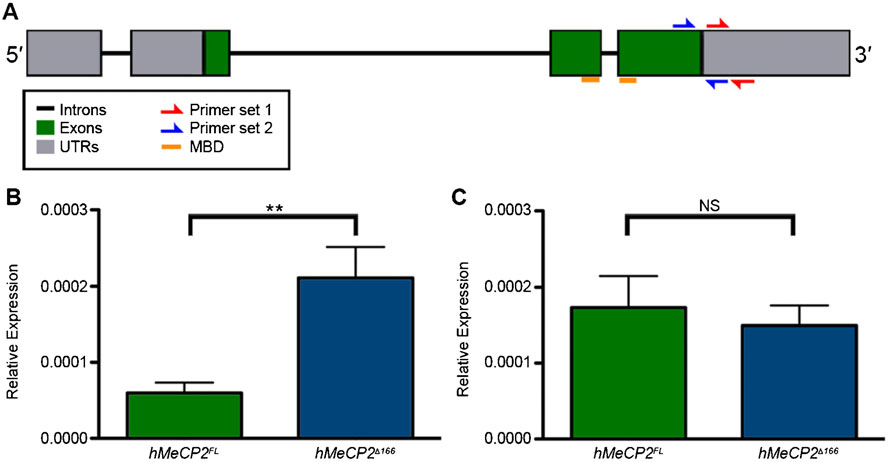
*hMeCP2^FL^* transcript levels are reduced compared to *hMeCP2*^Δ166^ levels. (A) Primer locations depicted in a schematic representation of *hMeCP2* locus. (B) For semi-quantitative RT-PCR experiments, RNA from the heads of adults expressing hMeCP2^FL^ in astrocytes *(alrm-Gal4/+;alrm-Gal4/UAS-hMeCP2^FL^*, green column), and hMeCP2^Δ166^ in astrocytes *(alrm-Gal4/UAS-hMeCP2^Δ166^;alrm-Gal4/+*, blue column). Adult *alrm-Gal4;UAS-hMeCP2^FL^* flies at 5–10 days post-eclosion showed significant decreases in *hMeCP2* transcript levels as compared to age-matched *alrm-Gal4;UAS-hMeCP2^Δ166^* adults (Mann–Whitney test, **P<0.05). (C) In comparison, transcript levels were not statistically different in adults expressing *hMeCP2^FL^* in octopamine neurons (*Tdc2-Gal4;UAS- hMeCP2^FL^*, green column) compared to age matched controls expressing the *hMeCP2^Δ166^* in octopamine neurons (*Tdc2-Gal4/UAS-hMeCP2^Δ166^*, blue column) (Mann–Whitney test, *P>0.7*). Reactions were performed in quadruplicate for each gene. Rpl32 expression was used as the reference control to normalize expression between treatment groups (error bars indicate s.e.m.).

To ask if the potential translational or transcriptional regulation of hMeCP2 could be overcome by providing more than one transgenic copy of *hMeCP2*, progeny were generated that contained two *alrm-Gal4* copies and two hMeCP2 transgenes. hMeCP2 expression in central brain astrocytes remains undetectable in the resulting progeny *(alrm-Gal4/UAS-hMeCP2^R106W^;alrm-Gal4/UAS-hMeCP2^FL^)* (supplementary material Fig. S4D,E). To examine *hMeCP2* transcript levels directly, total RNA was extracted from the heads of adults expressing hMeCP2^FL^ (*alrm-Gal4;alrm-Gal4/UAS-hMeCP2^FL^*) or hMeCP2^Δ166^ (*alrm-Gal4/UAS-hMeCP2^Δ166^;alrm-Gal4*). We performed quantitative RT-PCR experiments with primer sets directed to the last exon or 3′ UTR (Fig. 5A). Our results demonstrate that the levels of full-length hMeCP2 transcripts in astrocytes are significantly reduced when compared to *hMeCP2^Δ166^* transcript levels (Fig. 5B). In contrast, transcript levels do not change in adults expressing *hMeCP2^FL^* versus *hMeCP2^Δ166^* in amine neurons (Fig. 5C). Results from these experiments indicate that the absence of detectable hMeCP2^FL^ protein in a subset of adult stage astrocytes is likely due to temporally and spatially-controlled transcript degradation. Furthermore, the continued expression of hMeCP2^Δ166^ protein production identifies a region that possibly contains sites important for the targeting and degradation of *hMeCP2^FL^* transcripts.

## DISCUSSION

The importance of tightly controlling MeCP2 levels in the human nervous system has been underscored by numerous studies encompassing loss-of-function or overexpression conditions. Loss-of-function mutations in MeCP2 cause Rett Syndrome while duplications and/or triplications spanning the *MeCP2* locus result in progressive neurological disorders characterized by autism, motor abnormalities, and seizures (Ben-Shachar et al., 2009; del Gaudio et al., 2006; Ramocki et al., 2009; Ramocki et al., 2010; Van Esch et al., 2005). As the discovery of distinct neuronal and non-neuronal cell-types that contribute to the various MeCP2-related clinical phenotypes increases, the number of molecular targets of MeCP2 increases as well. Considering the critical role of MeCP2 not only during development but also in maintaining cellular function in adulthood (Castro et al., 2013; Garg et al., 2013; Luikenhuis et al., 2004; Nguyen et al., 2012; Olson et al., 2014), MeCP2 regulation whether by transcriptional control via DNA regulatory elements or post-transcriptional mechanisms by RNA–protein, RNA–RNA, or RNA–DNA interactions is likely to continue to expand in complexity and in importance (Singh et al., 2008).

Here we report on the spatial and temporal post-transcriptional regulation of human MeCP2 levels in *Drosophila* astrocytes and on circuit functional changes due to glial expression. Until relatively recently, astrocytes, along with other glia cell types, were believed to be structural cells that function to hold neurons together. It is now appreciated that astrocytes serve many functions, including developmental roles during synaptogenesis, maintenance of the extracellular environment and stabilization of cell–cell communications in the CNS. In addition, astrocytes are increasingly recognized as active partners in synaptic function including regulating basal synaptic transmission and synaptic efficacy leading to the proposal that normal brain output arises from the coordinated activity of a network comprising both neurons and glia (Eyo and Dailey, 2013; McGann et al., 2012; Navarrete et al., 2013; Perea and Araque, 2010; Sloan and Barres, 2014). A group of recent studies highlight the role of astrocytes in the modulation of circuit concerned with sleep and sleep-related rhythmogenesis (Florian et al., 2011; Halassa et al., 2009; Schmitt et al., 2012). Glial cells regulate slow oscillations, a specific thalamocortical activity that characterizes non-REM sleep, and sleep-associated behaviors (Fellin et al., 2012; Halassa et al., 2010). In our studies, hMeCP2 expression in *Drosophila* astrocytes caused a significant decrease in sleep with the reduction occurring at a specific time point, immediately after the day:evening transition. How potential cell or non-cell autonomous morphological or functional defects cause the distinct deficit in sleep observed in our *Drosophila* males expressing hMeCP2 in astrocytes is presently unknown. However, a recent study determined dendritic structure defects in motor neurons are caused by MeCP2 expression (Vonhoff et al., 2012) and as the neurons and circuits that regulate sleep duration, initiation, and maintenance are well studied in *Drosophila*, further analysis of these described sleep deficits should prove fruitful. Results from our sleep paradigm can also be viewed as the endpoint behavioral representation of synaptic connectivity and dysfunction of circuits in general, which is a fundamental theme in neurodevelopmental syndromes including RTT (Shepherd and Katz, 2011).

Although *MeCP2* is ubiquitously transcribed, the expression of distinct MeCP2 isoforms is developmentally regulated and heterogeneous in neuronal subpopulations and may be impacted by DNA methylation patterns at *MeCP2* regulatory elements may impact the differential expression of *MeCP2*/MeCP2 isoforms in brain regions (Olson et al., 2014). A series of separate studies suggest a role of RNAi-induced down-regulation of MeCP2 expression (Jin et al., 2008). In our studies, human MeCP2e2 and RTT R106W transcript levels significantly decrease in astrocyte subsets, while expression of the *MeCP2^Δ166^* allele is not altered. The region absent in MeCP2^Δ166^ contains the MBD domain, a nuclear localizing signal, and a region designated HMG1, due to the amino acid composition similarity to high mobility group (HMG) proteins (Adams et al., 2007; Adkins and Georgel, 2011; Becker et al., 2013; Ghosh et al., 2010). What types of evolutionarily conserved *cis*-regulatory elements are located in this area of the hMeCP2-coding region? Several studies have identified microRNA target sites, AU-rich elements, and G-quadruplexes within the transcribed regions of *hMeCP2* (Bagga and D'Antonio, 2013; Saunders et al., 2010). Many of these DNA regions influence DNA replication, transcription, and epigenetic mechanisms (Baral et al., 2013; Belotserkovskii et al., 2013). For example expression of the microRNA, miR-483-5p, decreased MeCP2 mRNA levels through the human-specific binding site in the MeCP2 long 3′ UTR (Han et al., 2013). At this point, our results suggest an endogenous factor expressed in *Drosophila* glia, targets a regulatory component or components located within the first 498 base pair region of hMeCP2. As wildtype MeCP2 levels in glial cells are essential for proper development and maturation of the brain, identifying cell-type specific mechanisms that activate or repress normal levels to achieve a controlled balance of MeCP2 expression would be useful in therapeutic considerations.

## MATERIALS AND METHODS

The following strains were used in this study: *alrm-Gal4#2*, *alrm-Gal4#3* (Doherty et al., 2009), *EAAT1-Gal4* (BL 8849), *dTdc2-Gal4* (Cole et al., 2005), *OmbC-Gal4* (Hofmeyer et al., 2008), *UAS-MeCP2^FL^* (Cukier et al., 2008), *UAS-MeCP2^R106W^* (Cukier et al., 2008), *UAS- hMeCP2^Δ166^* (Cukier et al., 2008), *UAS-Red Stinger* (BL 8545 and BL 8546), *UAS-nucLacZ* (BL 3955), *hs-flp* (BL 8862), *UAS-mCD8:GFP* (BL 5130), *20XUAS-6XGFP-Myc* (BL 52262), and *UAS>stop>mCD8:GFP* (BL 5007) from the Bloomington Stock Center, Bloomington, IN.

### Immunohistochemistry

Brains in Figs 1 and 2 were dissected according to the following protocol. Brain samples were dissected in phosphate-buffered saline (PBS) and fixed in 4% paraformaldehyde for 30 min at room temperature. Then, the brain samples were incubated in PBS containing 1% Triton X-100 and 10% normal goat serum (PBS-T) and degassed in a vacuum chamber to expel tracheal air with four cycles (depressurize to ∼70 mmHg then hold for 10 min). Next, the brain samples were blocked and penetrated in PBS-T at 4°C overnight and then incubated in PBS-T containing 1:50 mouse 4F3 anti-discs large monoclonal antibody (Developmental Studies Hybridoma Bank, University of Iowa) and 1:200 rabbit anti-MeCP2 (Cell Signaling D4F3) at 4°C for two days. After washing in PBS-T three times, the samples were incubated in PBS-T containing 1:250 biotinylated goat anti-mouse IgG (Molecular Probes) and 1:500 Alexa Fluor 488 goat anti-rabbit IgG (Molecular Probes) at 4°C overnight. Next, brain samples were washed and incubated with 1:500 Alexa Fluor 635 streptavidin (Molecular Probes) at 4°C overnight. Finally, after extensive washing, the immunolabeled brain samples were directly cleared in FocusClear (CelExplorer, Taiwan) for 5 min and then mounted in a drop of MountClear (CelExplorer) and then imaged under a Zeiss LSM 510 confocal microscope. Each cell body labeled by both Red Stinger and Alexa Fluor 488 in the central brain was manually marked with a landmark sphere. The total number of landmark spheres placed in the central brain was automatically counted with Amira (Visage Software, San Diego, CA).

Brains in all other figures were dissected according to a separate protocol. Adult male and female dissected brains were fixed in 4% paraformaldehyde (Electron Microscopy Sciences) for 25 minutes and labeled using a modification of protocols previously described (Certel et al., 2007). The following primary antibodies were used: rabbit anti-MeCP2 (1:200, Cell Signaling D4F3), mouse anti-MeCP2 (1:1000, Abcam AB50005), rat anti-CD8 (1:100, Molecular Probes), rabbit polyclonal anti-β-galactosidase (1:100, Abcam), mouse anti-β-galactosidase (1:50, Developmental Studies Hybridoma Bank), mouse 8D12 anti-repo (1:20, DSHB), anti-bruchpilot (mAb nc82, 1:30, Developmental Studies Hybridoma Bank) (Hofbauer et al., 2009), and monoclonal rabbit anti-GFP (1:200, Molecular Probes). Secondary antibodies include Alexa Fluor 488-conjugated goat anti-rabbit, Alexa Fluor 488-conjugated goat anti-rat Alexa Fluor 594-conjugated donkey anti-mouse, Alexa Fluor 594-conjugated goat anti-rabbit, Alexa Fluor 647-conjugated donkey anti-mouse (Molecular Probes), Images were collected on an Olympus Fluoview FV1000 laser scanning confocal mounted on an inverted IX81 microscope and processed using ImageJ (NIH) and Adobe Photoshop (Adobe, CA).

### qPCR

Total RNA from ∼50 combined heads was isolated by Tri-Reagent, (Molecular Research Center, Cincinnati, OH) following manufacturers protocols. RNA samples were treated using PerfeCTa DNase I kit (Quanta Biosciences, 95150-01K) according to manufacturer's protocols. RNA concentrations were subsequently measured with a ND-1000 nanodrop spectrometer. Reverse transcription was accomplished using Qscript cDNA supermix (Quanta Biosciences, 95048) according to manufacturer's protocols with 200 ng RNA in 10 µL. qPCR reactions were carried out using 5 µL PerfeCTa SYBR Green SuperMix for iQ (Quanta Biosciences, 95053-100), 4 µL cDNA diluted 1:5 after reverse transcription, primers at 5×10^−5^ ng/µl, and DEPC water to a total volume of 10 µL.

qPCR reactions were carried out using 5 µL PerfeCTa SYBR Green SuperMix for iQ (Quanta Biosciences, 95053-100), 80 ng cDNA, primers at 5×10^−5^ ng/µl, and DEPC water to a total volume of 10 µL. Reactions were carried out on an Agilent Stratagene Mx3005P platform. Samples were first held at 95°C for 10 minutes before forty cycles of 30 seconds at 95°C, 60 seconds at 52°C, and 60 seconds at 72°C. Reactions were completed with a final cycle of 60 seconds at 95°C, 30 seconds at 55°C, and 30 seconds at 95°C. Reactions were performed in quadruplicate for each gene and genotype. Expression of *ribosomal protein L32 (RpL32)* was used as the reference control to normalize expression between genotypes. Expression levels were calculated using the ΔΔC_T_ method. Primers used to amplify *RpL32* were F: 5′ATGCTAAGCTGTCGCACAAATG3′ and R: 5′GTTCGATCCGTAACCGATGT3′. To ensure validity of results, each qPCR experiment was repeated with different sets of *MeCP2* primers. The first set *MeCP2* primers was F: 5′TTAGTCCCTCAAGCCACCAG3′ and R: 5′GGACGGAGGAAGGGAAAGAA3′. The second set of *MeCP2* primers was F: 5′AACAGAGAGGAGCCTGTGGA3′ and R: 5′-ACTTCTGGCCCTGGTTAGGT3′.

### Sleep assay

Male pupae were isolated and aged individually for 2–3 days in 16×100 mm borosilicate glass tubes containing standard culture medium described above. During development and post-eclosion, progeny were entrained to standard L:D conditions, 12-hr light phase followed by a 12-hr dark phase, at 25°C and 50% relative humidity. Male adults, age 3–5 days post-eclosion, were transferred to individual 65×5 mm glass tubes (Trikinetics) containing 15 mm food and a cotton plug on either end. Flies were allowed a 24-hr period to recover from the CO_2_ anesthetization necessary for tube transference. The locomotor activity patterns of individual experimental and control males were recorded by the *Drosophila* Activity Monitoring (DAM) system (Trikinetics) for a period of 10 consecutive days using the 1-min bin acquisition mode. Data from the first and last day were removed. The resulting activity data was analyzed using the Counting Macro 5.19.5 (CM) program (R. Allada, Northwestern University, Evanston, IL). Sleep related parameters including amount of sleep, latency to sleep, number of sleeping bouts, length of each bout and waking activity etc. were measured (Pfeiffenberger et al., 2010). A sleep bout was defined as complete inactivity for a period of 5 consecutive minutes as in previous studies (Shaw et al., 2000). Graphpad Prism and Adobe Photoshop were used to generate graphs.

### Statistics

Bootstrap-based resampling procedures v1.3 were used to quantify the effect of hMeCP2 expression in astrocytes on sleep during ZT12-15 (Howell, 2002). Average amount of sleep per 30 min bin was extracted from sleep eduction data for ZT12-15. Sample distribution was empirically determined by random sampling of residuals with replacement and *F-statistic* was computed for each of the 50,000 bootstrapped residuals. The resulting distribution was used to evaluate the likelihood of obtaining an F-statistic greater than the value obtained from the sample means at 95% confidence interval. Results were cross-validated with permutation tests that involve randomization without replacement.

## Supplementary Material

Supplementary Material
