# Supplementary material for: Astrocyte-specific regulation of hMeCP2 expression in Drosophila
Source: Biol Open. 2014 Oct 10;3(11):1011–9. doi: 10.1242/bio.20149092 (PMC4232758; doi:10.1242/bio.20149092)
Supplement: Supplementary Material [file supp_3_11_1011__index.html]

Astrocyte-specific regulation of hMeCP2 expression in Drosophila — Supplementary Material 

# Astrocyte-specific regulation of hMeCP2 expression in *Drosophila*

## bio.20149092 Supplementary Material

**Files in this Data Supplement:**

- Supplementary Material - David L. Hess-Homeier et al. doi: 10.1242/bio.20149092
