# Supplementary material for: Astrocyte-specific regulation of hMeCP2 expression in Drosophila
Source: Biol Open. 2014 Oct 10;3(11):1011–9. doi: 10.1242/bio.20149092 (PMC4232758; doi:10.1242/bio.20149092)
Supplement: Supplementary Material [file supp_bio.20149092_bio.20149092-s1.pdf]

Biology Open (2014) 000, 1–9 doi:10.1242/bio.20149092

Supplementary Material

David L. Hess-Homeier et al. doi: 10.1242/bio.20149092

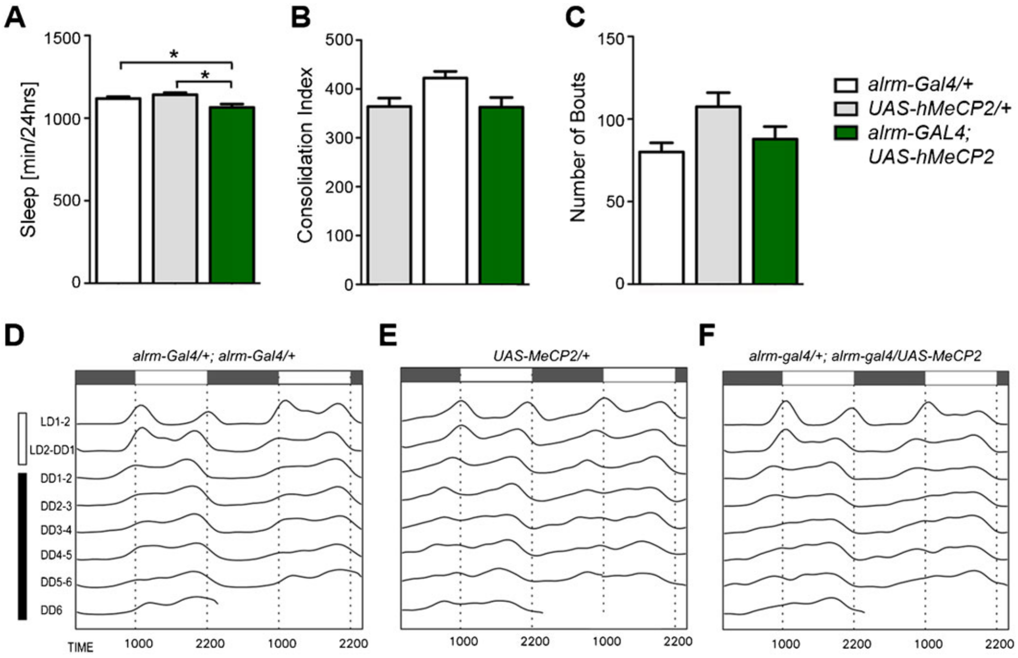

Fig. S1. hMeCP2 expression in astrocytes does not alter multiple sleep parameters. (A–C) The amount of sleep, sleep patterns, and sleep fragmentation was quantified in individual adult males expressing MeCP2 in astrocytes (*alrm-Gal4;UAS-hMeCP2)*. (A) The total amount of sleep exhibited a small but significant reduction between controls (*alrm-Gal4/+* and *UAS-hMeCP2*^{FL}*/+*) vs. experimental *(alrm-Gal4;UAS-hMeCP2*^{FL}*)* males. *(*$F(2,115)$*: 6.384,* $P=0.0023$*; one-way ANOVA with Holm–Sidak's multiple comparison test*. (B,C) Significant effects were not observed in pairwise comparison of control vs. experimental groups measuring: (B) the average weighted length of each bout as a weighted consolidation index (C.I.) *(p(adj, UAS-hMeCP2*^{FL}*/+ vs. alrm-Gal4;UAS-hMeCP2*^{FL}*)*>0.9 and *p(adj, alrm-Gal4/+ vs. alrm-Gal4;UAS-hMeCP2*^{FL}*)*$=0.0316$*; Kruskal–Wallis test with Dunn's multiple-comparison)*, and (C) the total number of sleep bouts *(P(adj, UAS-hMeCP2*^{FL}*/+ vs. alrm-Gal4;UAS-hMeCP2*^{FL}*)*$=0.1659$ and *P(adj, alrm-Gal4/+ vs. alrm-Gal4;UAS-hMeCP2*^{FL}*)*$=0.7423$*; Kruskal–Wallis test with Dunn's multiple-comparison). adj: multiplicity adjusted P-value for each comparison*. (D–F) Double-plotted actograms representing day-wise distribution of group locomotor activity plotted in 30-min intervals in controls (D,E) and experimental males (F). LD1 corresponds to the first day of activity monitoring of flies entrained to 12 hr LD cycles for at least 3 days. On the third recorded day, controls and experimental flies were switched to constant darkness (DD) conditions. (F) Males expressing hMeCP2^{FL} in astrocytes (*alrm-Gal4;UAS-hMeCP2*^{FL}) maintain both activity bouts with a reduced amplitude.

Table S1. Average activity distribution in free-running conditions of *hMeCP2*^{FL}-expressing flies

Genotype	N	Rhythmic (Nr)	Rhythmic (%)	Period (Tau) (hr ± SEM)	Power (mean ± SEM)
UAS-hMeCP2FL/+	31	31	100	23.9±0.08	145.9±6.91
alrm-Gal4/+; alrm-Gal4/+	24	23	95.8	24.1±0.14	107.7±3.54
alrm-Gal4/+; alrm-Gal4; UAS-hMeCP2FL	21	20	95.2	23.7±0.11	89.9±6.39

3–5 day old adult males from controls (*UAS-hMeCP2*^{FL}*/+, alrm-Gal4/+*) and experimental (*alrm-Gal4;UAS-hMeCP2*^{FL}) groups were analyzed for changes in rhythmicity, period, and average activity profiles under free-running or constant darkness (DD) conditions.

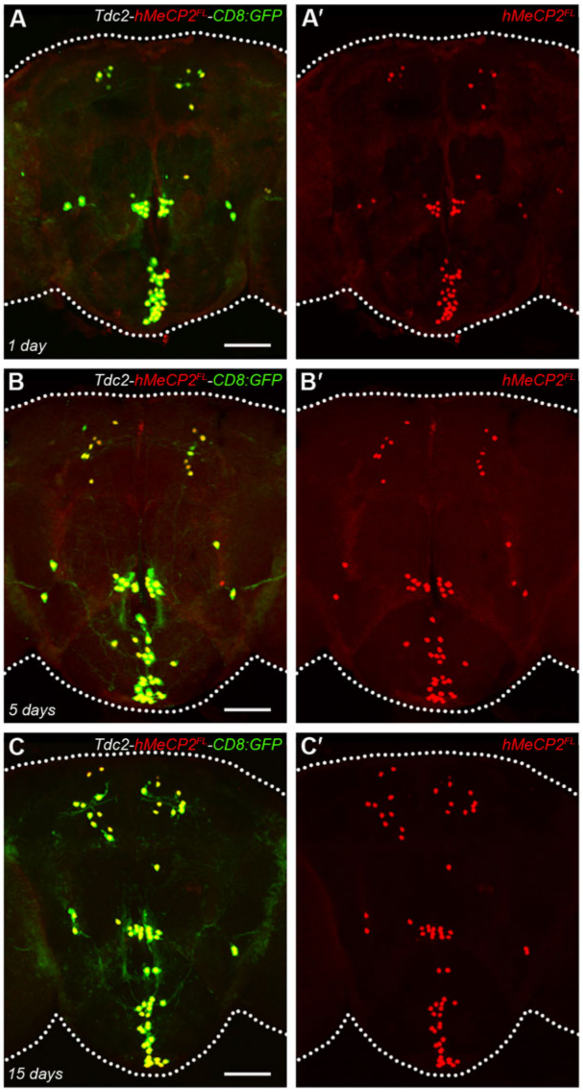

Fig. S2. Transgenic hMeCP2 expression is maintained in OA neurons. (A–C) hMeCP2 expression in octopamine neurons does not change at 1, 5, or 15 days post-eclosion (*Tdc2-Gal4/UAS-CD8:GFP;UAS-hMeCP2* progeny). (A′–C′) hMeCP2 expression is detected by immunofluorescence with the mouse hMeCP2 antibody (red, Abcam). Scale bar represents 50 µm.

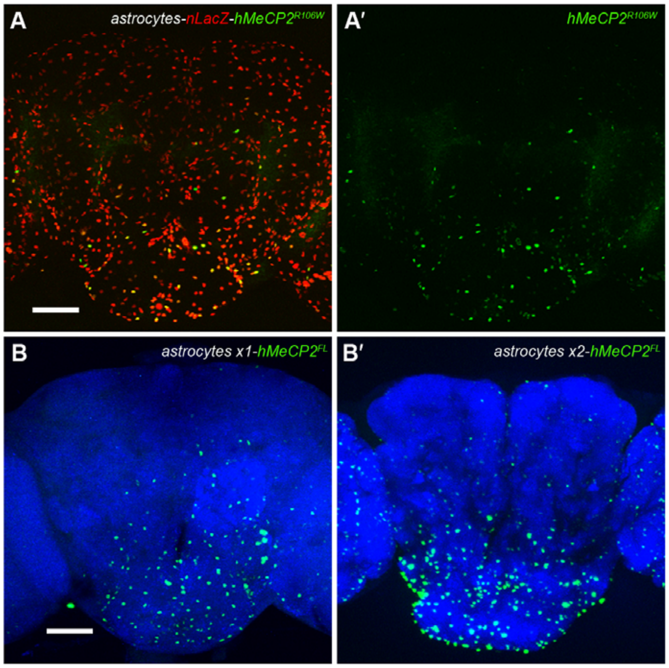

Fig. S3. Transgenic hMeCP2 expression is not dependent on transgenic copy number or antibody penetration. (A,A′) LacZ production is visible in astrocytes throughout the *Drosophila* central brain in experimental adults expressing hMeCP2^{R106W} and nucLacZ *(alrm-Gal4;UAS-nucLacZ/UAS-hMeCP2^{R106W})* (red, anti-β-gal, Developmental Hybridoma Bank; green, hMeCP2, Cell Signaling). **(B,B′)** The reduction in hMeCP2^{FL} occurs with one transgenic copy of *alrm-Gal4* driver (B) or two (B′). Scale bars represent 50 µm.

Biology Open (2014) 000, 1–9 doi:10.1242/bio.20149092

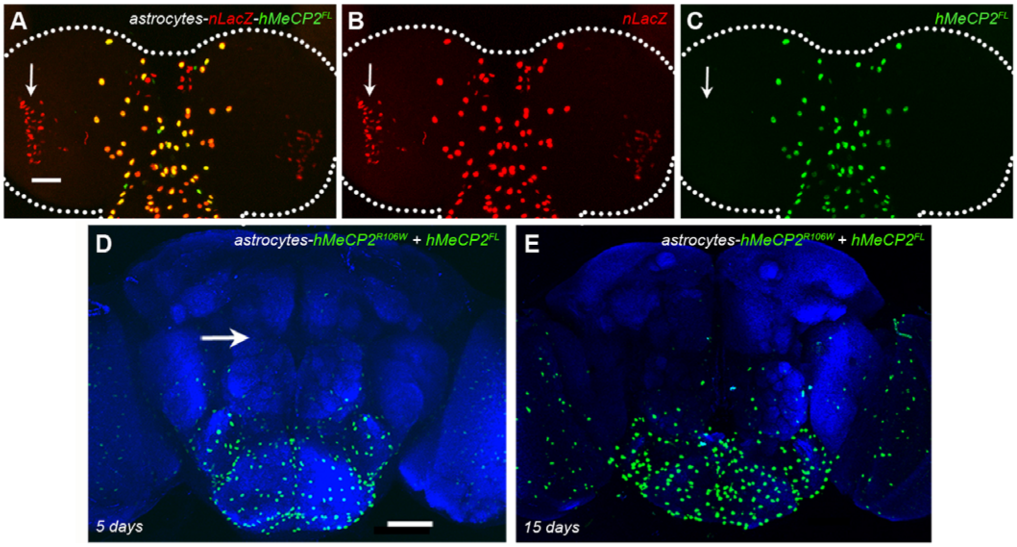

Fig. S4. Astrocyte subsets reduce hMeCP2^{FL} expression in larval stages and with increased transgenic copies. (A–C) Astrocytes within the central brain of third instar larvae co-localize β-gal and hMeCP2 in *alrm-Gal4;UAS-nucLacZ/UAS-hMeCP2^{FL}* progeny. An absence of hMeCP2 expression is observed in astrocytes located in the optic lobe (arrows). **(D,E)** Dissected brains from progeny containing copies of the *UAS-hMeCP2^{FL}* and the *UAS-hMeCP2^{R106W}* transgenes driven by *alrm-Gal4* show a reduction of hMeCP2 expression at later stages of adulthood (arrow). Scale bars represent 50 µm.

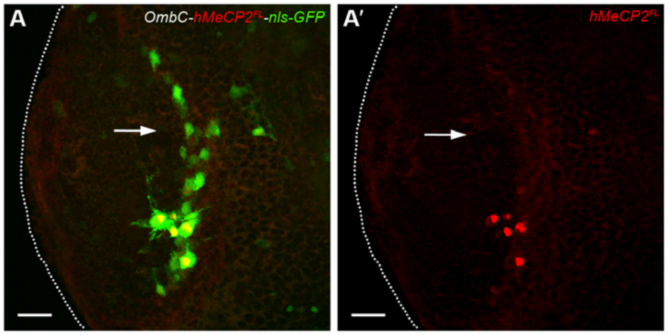

Fig. S5. Transgenic hMeCP2^{FL} expression is reduced in subsets of inner-optic chiasm giant glia (ICg-glia). (A,A′) Left optic lobe of late 3rd instar larval CNS co-expressing hMeCP2^{FL} and nuclear GFP in ICg-glia (*UAS-nlsGFP;UAS-MeCP2^{FL}/OmbC-Gal4*). hMeCP2^{FL} expression is absent in a subset of ICg-glia that express nuclear GFP. **(A′)** hMeCP2^{FL} expression is detected by immunofluorescence with the mouse hMeCP2 antibody (red, Abcam). Scale bar represents 20 µm.

Biology Open
